# Methylphenidate abuse and misuse in patients affected with a psychiatric disorder and a substance use disorder: a systematic review

**DOI:** 10.3389/fpsyt.2024.1508732

**Published:** 2024-11-18

**Authors:** Stefania Chiappini, Pietro Domenico Gramuglia, Alessio Mosca, Clara Cavallotto, Andrea Miuli, John Martin Corkery, Amira Guirguis, Fabrizio Schifano, Giovanni Martinotti

**Affiliations:** ^1^ Saint Camillus International University of Health Sciences, School of Medicine, Rome, Italy; ^2^ Department of Neuroscience, Imaging and Clinical Sciences, “G. D’Annunzio” University, Chieti, Italy; ^3^ Psychopharmacology, Drug Misuse and Novel Psychoactive Substances Research Unit, School of Life and Medical Sciences, University of Hertfordshire, Hatfield, United Kingdom; ^4^ Swansea University Medical School, Swansea University, Swansea, United Kingdom

**Keywords:** methylphenidate, MPH, ADHD, dual diagnosis, SUD, drug misuse, pharming

## Abstract

**Background:**

Methylphenidate (MPH), a central nervous system stimulant primarily prescribed for attention-deficit/hyperactivity disorder (ADHD), has seen increasing rates of misuse and abuse, particularly in patients with dual diagnosis (co-occurring psychiatric disorders and substance use disorders/SUDs). The heightened risk of dependence and adverse effects in these vulnerable populations warrants a systematic review to assess the prevalence and pattern of abuse/misuse of MPH among patients within this population and to understand potential risk factors, patterns of misuse, and outcomes, including the impact on psychiatric symptoms and overall mental health, the effects on SUD (e.g., exacerbation or mitigation of symptoms), and the incidence of adverse events and complications (e.g., cardiovascular issues, psychological effects).

**Methodology:**

A systematic review was conducted in August-September 2024 using both PubMed and Scopus databases. The following search strategy was used: TITLE-ABS-KEY (methylphenidate OR Ritalin OR Concerta) AND TITLE-ABS-KEY (abuse OR misuse OR dependency OR addiction) AND TITLE-ABS-KEY (dual diagnosis OR comorbid psychiatric disorder OR psychiatric disorder AND substance use disorder). The systematic review was structured in accordance with the PRISMA guidelines and identified studies were assessed by title/abstract and full text screening against eligibility criteria.

**Results:**

A total of 12 studies were selected for analysis after screening for relevance, quality, and adherence to inclusion criteria. Findings indicated that individuals with psychiatric disorders, particularly conduct disorder (N=593/1551 individuals), mood disorder (N=90/1551 individuals), anxiety disorder (N=66/1551 individuals), personality disorder (N=44/1551 individuals) and major depression disorder (N=40/1551 individuals), were more likely to misuse MPH. Co-occurring SUD, especially involving Alcohol Use Disorder (N=475/1551 individuals), Cannabis Use Disorder (N=371/1551 individuals), Nicotine Use Disorder (N=343/1551 individuals), Cocaine Use Disorder (N=68/1551 individuals), significantly elevated the risk. Misuse often involved higher doses than prescribed (N=84/1551 individuals) or using non-oral routes of administration (N=20/1551 individuals; e.g., snorting). Adverse outcomes included heightened risk of gastrointestinal events (N=201/1551 individuals), cardiovascular events (N=108/1551 individuals), psychosis (N=69/1551 individuals), and exacerbation of psychiatric symptoms (N=1082/1551 individuals).

**Conclusion:**

MPH misuse and abuse are significant concerns in patients with psychiatric disorders and SUD. Risk factors include impulsivity, history of substance abuse, and access to prescription stimulants. Integrated therapeutic approaches and stricter prescription monitoring are recommended to mitigate misuse risks.

**Systematic review registration:**

https://www.crd.york.ac.uk/prospero/, identifier CRD42024576724.

## 1 Introduction

### 1.1 Methylphenidate: overview and indications

Methylphenidate is a central nervous system (CNS) stimulant primarily used in the treatment of attention deficit hyperactivity disorder (ADHD) and narcolepsy. It functions by inhibiting the reuptake of dopamine and norepinephrine, leading to increased concentrations of these neurotransmitters in the brain, which enhances attention, focus, and impulse control (1, 2). According to the European Medicines Agency (EMA), methylphenidate is indicated for the treatment of ADHD in children and adolescents aged 6 years and older. It is generally recommended as part of a comprehensive treatment program that includes psychological, educational, and social measures. In some cases, it may also be prescribed for adults with ADHD, although this is less common (1). Methylphenidate may also be prescribed off-label for narcolepsy (1). In the United States (US) the Food and Drug Administration (FDA) has approved methylphenidate for the treatment of ADHD in children aged 6 and older, adolescents, and adults. It is typically used as part of a broader treatment strategy, including behavioural therapy and other interventions (2). In the US, it is also approved for the treatment of narcolepsy in adults, helping to manage symptoms of excessive daytime sleepiness (2).

### 1.2 Methylphenidate formulations: overview and key differences

Methylphenidate is available in various formulations, each designed to optimize the pharmacokinetic profile, enhance patient adherence, and tailor the therapeutic effect to individual needs. The differences in these formulations primarily relate to their release mechanisms, duration of action, and bioavailability: I) Immediate-release (IR) methylphenidate formulations, such as Ritalin^®^ IR, provide a rapid onset of action with a relatively short duration, typically lasting 3-4 hours. They are often administered multiple times a day (usually two to three times) to maintain therapeutic effects; these formulations are beneficial for patients who require flexible dosing or fine-tuning of dose throughout the day (3). II) Sustained-release (SR) and extended-release (ER) formulations, such as Ritalin SR^®^, Concerta^®^, Metadate CD^®^, and Quillivant XR^®^, are designed to extend the duration of action to 8-12 hours, reducing the need for multiple daily dosing. These formulations are favoured for their ability to maintain stable plasma concentrations throughout the day, which helps reduce the likelihood of peak-trough fluctuations that can lead to side effects or suboptimal symptom control. This also improves patient adherence by simplifying dosing regimens to once daily (4). Moreover, there are non-traditional formulations that provide ER methylphenidate in orally disintegrating tablet (ODT) or liquid forms and are particularly used useful in paediatric populations or individuals with difficulties swallowing tablets. III) Finally, there are available on the market transdermal patches that provide continuous release of methylphenidate over a 9-hour wear time: the onset of action is slower compared to oral formulations, but they offer the advantage of being removed if adverse effects occur, thereby controlling the duration of exposure (4).

### 1.3 Methylphenidate misusing issues

Consistently with an increasing prescription of methylphenidate, closely tied to rising ADHD diagnoses, its abuse and misuse are a growing concern (5, 6), particularly among patients with dual diagnosis—those with concurrent psychiatric disorders and substance use disorders (SUD) (7–10). This is supported by findings recording the appearance of methylphenidate on the illicit market (11). Current literature available demonstrated varying prevalence rates of methylphenidate misuse among patients with dual diagnosis, often higher than in the general population (12). Reasons why it could be misused by students and young adults may include cognitive enhancement or recreational purposes (13–15). A systematic review by Kaye and Darke (2012) (16) reported that up to 25% of individuals with psychiatric disorders and concurrent SUD misused prescription stimulants, including methylphenidate. Another study by Levin et al. (2008) (17) found that approximately 30% of patients with ADHD and co-occurring SUD misused their prescribed stimulants. Research indicates that individuals with dual diagnosis are at an elevated risk for methylphenidate misuse, especially for those agents with pharmacologic and pharmacokinetic characteristics that provide a rapid high, primarily due to the overlapping features of ADHD and SUD, such as impulsivity and sensation-seeking behaviours (7, 18, 19).

Methylphenidate’s abuse liability is closely tied to its pharmacological action on the dopaminergic system, specifically its ability to inhibit the reuptake of dopamine (DA) and norepinephrine (NE) by binding to their respective transporters, particularly the dopamine transporter (DAT) and the norepinephrine transporter (NET) (20–22). This leads to an accumulation of dopamine in the synaptic cleft, which enhances dopaminergic neurotransmission. The dopaminergic pathways most affected by methylphenidate include the mesolimbic and mesocortical pathways, which are associated with reward, motivation, and executive function (21, 22).

The main aim of the study was: understanding the prevalence and pattern of abuse/misuse of methylphenidate among patients with dual diagnosis (concurrent psychiatric disorder and SUD), and what are the associated clinical outcomes and risk factors.

## 2 Methodology

Systematic electronic searches were performed from August to September 2024 on PubMed and Scopus databases. The following search strategy was used: TITLE-ABS-KEY (methylphenidate OR Ritalin OR Concerta) AND TITLE-ABS-KEY (abuse OR misuse OR dependency OR addiction) AND TITLE-ABS-KEY (dual diagnosis OR comorbid psychiatric disorder OR psychiatric disorder AND substance use disorder). Moreover, other relevant papers not resulting from the described searches were added from references of included articles. The systematic review was structured in accordance with the PRISMA (23, 24) guidelines and identified studies were assessed by title/abstract and full text screening against eligibility criteria (see PRISMA checklist in **Supplementary Materials**).

The eligibility criteria included the selection of exclusively original articles written in English that provide data on the abuse/misuse of methylphenidate among patients with dual diagnosis. The data were collected in an Excel table containing the first author’s name and year of publication of the study, study design, demographic variables (gender, age, psychiatric history) and eventual details on the abuse/misuse of the drug (e.g., dosage and route of administration). The exclusion criteria for both selection phases were: 1) non-original research (e.g., review, metanalysis, commentary, editorial, letter to the editor without data available, and book chapter); 2) non-full-text articles (e.g., meeting abstract); 3) language other than English; 4) animal/*in vitro* studies; 5) articles not relating to abuse/misuse of methylphenidate and dealing with a specific diagnosis of SUD co-occurring with a psychiatric disorder. The research was registered on PROSPERO with the following ID number: CRD42024576724.

In addition to the main aim of the study, other specific outcomes to be measured and analysed include data on prevalence of abuse/misuse of methylphenidate in the target population, e.g., frequency, rates, patterns of use, etc., but also clinical outcomes, such as the impact on psychiatric symptoms and overall mental health, the effects on SUD (e.g., exacerbation or mitigation of symptoms), the incidence of adverse events and complications (e.g., cardiovascular issues, psychological effects), and eventual risk factors (e.g., demographic factors, psychiatric and SUD profiles, social and environmental factors contributing to misuse). The research question was formulated following the PICO framework guidelines (**Supplementary Materials Table 1**). The population (P) includes patients affected with a dual diagnosis (co-occurring psychiatric disorder and SUD) misusing and abusing methylphenidate. The intervention (I) focuses on the rate of methylphenidate abuse/misuse in this specific population of patients, while the comparison (C) is made with the general population. The outcome (O) encompasses an understanding of the prevalence and pattern of abuse/misuse of methylphenidate among patients with dual diagnosis, and what are the associated clinical outcomes and risk factors. The research question was: “What are prevalence and patterns of methylphenidate abuse or misuse among patients with dual diagnosis, and what are the associated risk factors and clinical outcomes?”. The assessment of risk of bias was made in accordance with the Cochrane risk of bias 2 (RoB 2) tool. The analysis of the risk of bias among the twelve articles provided valuable insights into the complexities of methylphenidate abuse within populations with dual diagnoses (**Supplementary Materials Table 2**). Selection bias was generally mitigated in many studies through the inclusion of diverse patient demographics, such as different age groups, gender representations, and varying psychiatric histories. Although some studies utilised convenience sampling, they often focused on specific populations known to exhibit higher rates of substance use, thereby enhancing the relevance of their findings within these contexts. Measurement bias was addressed in several studies by incorporating structured interviews and standardised assessment tools, which increased the reliability of the reported data on methylphenidate misuse. While some articles relied on self-reported data, this approach was frequently supplemented with corroborating clinical assessments, minimising the potential for underreporting. The acknowledgment of potential measurement limitations by the authors also indicates a commitment to transparency and scientific rigor. Reporting bias was not prominently observed, as the majority of studies presented comprehensive findings and discussed both positive and negative outcomes associated with methylphenidate misuse. This balanced reporting fosters a more nuanced understanding of the issue, contributing to a broader dialogue in the field. Confounding bias was effectively managed in many studies by accounting for various psychiatric comorbidities and substance use histories. Some articles utilised statistical methods to control for confounders, thus enhancing the validity of their conclusions regarding the relationship between methylphenidate abuse and underlying psychiatric disorders.

In conclusion, the overall assessment suggests a low to moderate level of bias across the analysed articles.

## 3 Results

**Figure 1:**
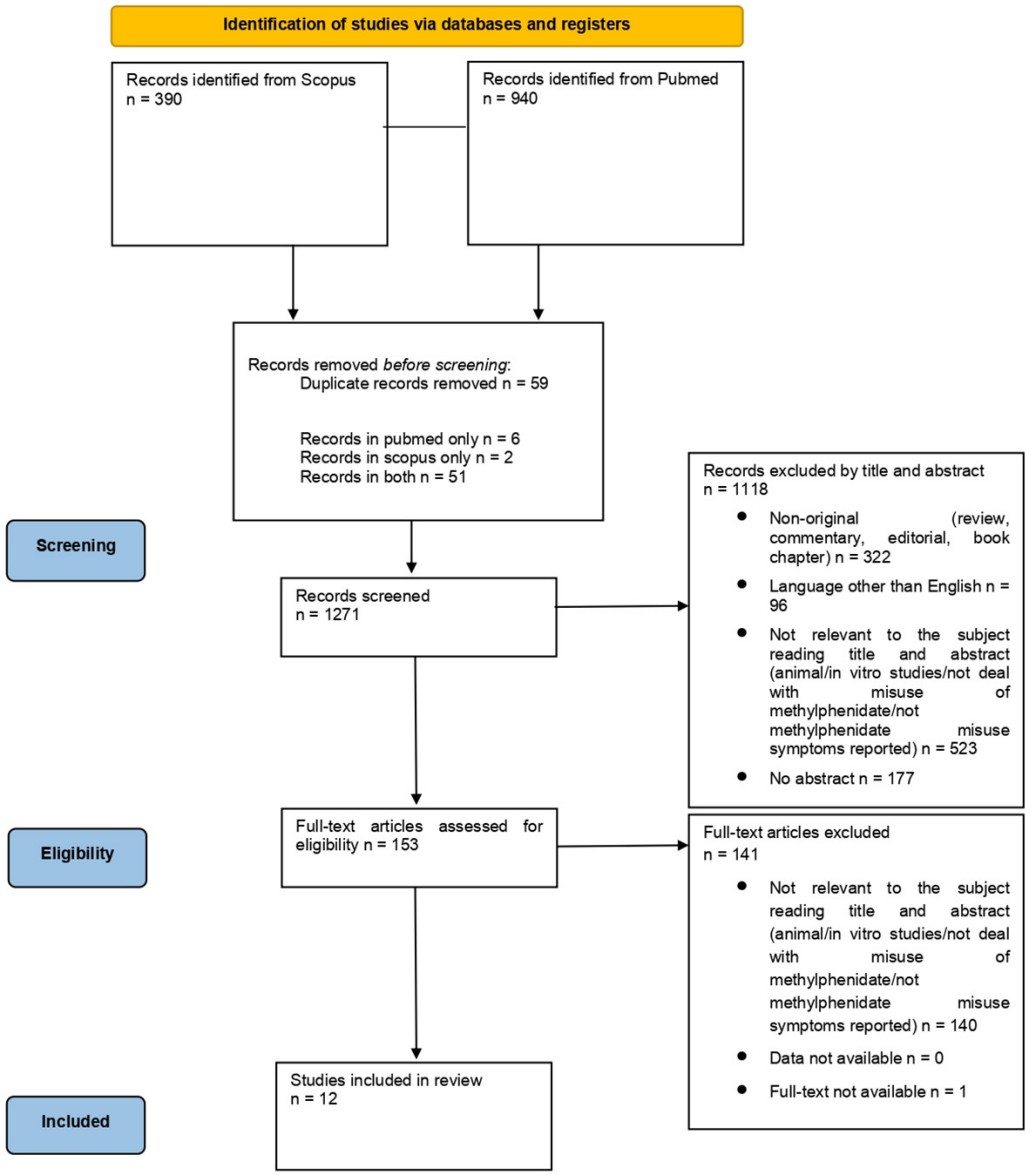
PRISMA 2020 flow diagram. Adapted from: The PRISMA 2020 statement: an updated guideline for reporting systematic reviews, by Page MJ, McKenzie JE, Bossuyt PM, Boutron I, Hoffman TC, Mulrow CD, et al., licensed under CC-BY 2.0, https://doi.org/10.1136/bmj.n71 (24).

From a total of 1,330 articles (PubMed = 940; Scopus = 390; other sources = 0), after deduplication (n = 59), a total of 1,271 records were screened. Among the articles screened, 523 were considered not relevant to the subject after reading the title and abstract, 96 were not written in English and 322 were non-original articles. Of the 153 full-text articles assessed for eligibility, 140 did not match the inclusion criteria for our review; finally, 12 articles were included in the systematic review (**Figure 1**).

Studies recorded were: one observational studies (N=1) (25), one case series (N=1) (26), three case reports (N=3) (27–29), three randomised controlled trials (N=3) (30–32), one survey (N=1) (33), one SPECT study (N=1) (34), one cross sectional study (N=1) (35), one longitudinal study (N=1) (36). A detailed summary of the 12 articles is included in **Table 1**.

**Table 1:** Overview of studies recording methylphenidate abuse and misuse in patients affected with a psychiatric disorder and a substance use disorder: summary of the main findings.

Study	Study design (country)	Sample features (gender, mean age ± SD)	Psychiatric diagnosis	Psychiatric therapy (if any)	Methylphenidate (dosage, ROA)	Substance Use Disorder	Clinical effects reported	Treatments reported (if any)	Notes
Tamm et al. (31)	Randomized Controlled Trial (USA)	N=299 16.5 yy (13-18) (SD=1.3)	ADHD, Conduct Disorder	OROS-MPH	The study included a 16-week active treatment phase Oral (18 mg/day was escalated during the first two study weeks to a maximum of 72 mg/day)	Alcohol Use Disorder/CUD	None	CBT	All participants were enrolled in concurrent outpatient substance treatment consisting of weekly individual CBT. OROS-MPH improved SUD outcomes in adolescents with comorbid conduct disorder compared to placebo. Comorbid conduct disorder predicted worse ADHD outcomes, there was an interaction effect for SUD outcomes, such that adolescents with conduct disorder who were prescribed OROS-MPH had significantly better substance use outcomes than adolescents with conduct disorder who received placebo. Moreover, adolescents with higher substance use severity showed poorer response outcomes: they had less reduction in ADHD symptom ratings and fewer negative urine drug screens
Konstenius et al. (30)	Randomized placebo-controlled 24-week double-blind trial (Sweden). This trial aimed at evaluating the effect MPH in doses up to 180 mg/day in patient with a co-diagnosis of ADHD and amphetamine dependence.	N=54, M 42 yy (18-65) subjects were randomized into two parallel groups (MPH or identical placebo). The medication started 14 days before release from prison (two participants started 3 days and one 5 days before release) and continued for 24 weeks	ADHD, Antisocial Personality Disorder (n=28)	OROS-MPH	MPH (doses up to 180 mg/day)	Stimulant Use Disorder (Amphetamine at urine toxicology)	One participant reported suicidal ideation at week 5 in treatment, at which point study medication was discontinued. High blood pressure, palpitations, muscular cramps, headache, abdominal discomfort, sleep problems, loss of appetite, depressed mood, sweating, fatigue, anxiety, dry mouth, craving, chest pain, restlessness, dizziness, auditory hallucinations, tics, agitation, suicidal ideation	Cognitive–behavioural therapy (once weekly)	The MPH-treated group reduced their ADHD symptoms during the trial (P = 0.011) and had a significantly higher proportion of drug-negative urines compared with the placebo group (P = 0.047), being MPH treatment hypothetically reducing craving and the risk for relapse to substance use
Winhusen et al. (32)	Randomized Controlled Trial (USA)	Datasets from two randomized placebo-controlled trials of OROS-MPH for the treatment of ADHD, one conducted on 303 adolescents (M=240; F=63), 16.5 yy (13-18) with at least one non-nicotine SUD and one on 255 adult smokers (M=144; F=110), 37.8 yy (18-55)	ADHD, Conduct Disorder (n=98), MDD (n=38)	OROS- MPH	OROS-MPH 18 mg/day up to 72 mg/day. In both studies, participants were randomized to OROS-MPH or matching placebo in a 1:1 ratio. Participants were given medication for 2 weeks to help ensure continuity of treatment	Non-nicotine SUD	Feel depressed (hopelessness sadness, emptiness), mood instability, craving (nicotine, alcohol or other drugs)	NA	The adolescent ADHD study included a 16-week active treatment and for weekly 1-hour individual CBT sessions. The adult ADHD study included an 11-week active treatment phase during which participants took OROS-MPH or placebo and were scheduled for weekly 10-minute individual smoking cessation counselling sessions. All participants in the adult ADHD study received transdermal nicotine patches. The adolescents also had a greater rate of co-occurring disorders, with 32.3% of the adolescents meeting criteria for conduct disorder and 12.5% meeting criteria for MDD. OROS-MPH participants experienced significantly more adverse events than placebo participants in both groups: in the adolescent, the adverse events reported are: Cardiac (n=9), Gastrointestinal (n=60), Metabolic (n=40), Nervous system disorder (n=107), Psychiatric (n=67); in the adult group: Cardiac (n=11), Gastrointestinal (n=65), Metabolic (n=33), Nervous system disorder (n=87), Psychiatric (n=140) Significantly more adolescents compared to the adults lost pills, regardless of treatment condition, which might indicate a greater likelihood for adolescents to misuse/divert their medication or might simply reflect greater carelessness on the part of the adolescents. Higher baseline use of alcohol and cannabis was associated with an increased risk of experiencing adverse events in OROS-MPH, relative to placebo, which suggests the need to monitor side-effects closely in substance abusing adolescents
Szobot et al. (34)	SPECT study (Brazil)	N=17, M 15-21 yy	ADHD, MDD (n=3), Conduct Disorder (n=13), Separation Anxiety Disorder (n=1), Oppositional Defiant Disorder (n=6)	NA	MPH Oral, 0.3 mg/kg/day at week 1; 0.7 mg/kg/day at week 2; and 1.2 mg/kg/day at week 3	CUD, Cocaine Use Disorder, Alcohol Use Disorder, Nicotine Dependence	NA	None	The magnitude of dopamine transporter (DAT) blockade induced by MPH in this population is similar to what is found in ADHD patients without SUD comorbidity. Significant correlation between MPH dosage and DAT occupation. After 3 weeks on MPH, there was a 52% reduction of dopamine transporter binding at the left and right caudate. Similar decreases were found at the left and right putamen The estimated MPH dose required to block half of the DAT was 0.25 mg/kg of oral MPH blockade of DAT at the striatum
Bron et al. (25)	Observational Study (Netherlands)	N=325 (M=157; F=168), 17-64 yy	ADHD (n=325), Anxiety disorder (n=66), Mood Disorder (n=89), Personality Disorder (n=8)	NA	IR-MPH (10-40 mg)	Nicotine Dependence (mean 10.5 mg, SD 8.5 vs. 12.3 mg, SD 9.5)	Increased nicotine craving after MPH use	None	Tobacco consumption increased with 1.3 cigarettes per day after three-months of MPH use
Vogel et al. (35)	Cross sectional study (Switzerland)	N=20 (M=15; F=5), 42.7 yy (29-54)	ADHD, Schizophrenic spectrum disorder (n=4), Affective Disorder (n=11), Post-traumatic Stress Disorder (n=3), Personality disorder (n=5)	NA	MPH: Oral (n=4)/Nasal(n=7)/IV(n=7) at the dosage of 20 up to 100 mg daily	Cocaine Abuse Disorder (n=13)	Loss of appetite, tachypnoea, insomnia, palpitations, nervousness, paranoia, anxiety, depression, fatigue, craving. Following use of MPH, 70% reported feeling relieved and satisfied, 60% mentioned increased alertness, energy, and activity, and 35% enhanced concentration.	Opioid maintenance treatment: Methadone/buprenorphine (n=17); slow-release oral morphine (n=3). Mean duration of opioid maintenance treatment episode was 5.8 years with a range of 2–13 years.	12 of the 20 patients received a prescription for MPH and abused it. Another 6 abused illicitly obtained MPH. Parenteral use was associated with a higher risk of complications such as cardiotoxicity and pneumotoxicity Additional substance used in the past month were recorded: Opioids (n=10), Cocaine (n=13), Alcohol (n=15), Sedatives (n=12), Nicotine (n=20)
Wilens et al. (36)	10-years Longitudinal study (USA)	N=98 (M=65; F=33), 20.8 yy ± 5. Among them, n=46 (48%) met diagnostic criteria for a SUD	ADHD (n=55), and Conduct Disorder (n=21)	In ADHD subjects: Selective Serotonin Reuptake Inhibitors (n=30)/Stimulants (n=94)/Benzodiazepines (n=4)/Lithium (n=10)/Tricyclic Antidepressants (n=20), Alpha-adrenergic agents (n=15), Neuroleptics (n=6). In Non-ADHD subject: Selective Serotonin Reuptake Inhibitors (n=78), Benzodiazepines (n=20), Atypical neuroleptics (n=20), Stimulants (n=10), Lithium (n=10)	OROS-MPH (Oral, NA)	Alcohol use disorder, Multiple Drug Abuse	None	None	All ADHD subjects diverting their medication had either comorbid conduct disorder or SUD. Indeed, the 11% of the ADHD subjects diverted their medication, 22% took too much or misused their prescribed medication, and 10% got “high” on their medication. All of the medications misused or diverted were the immediate-release preparations of stimulants.
Gordon et al. (33)	Survey (USA)	N=162 (M=104; F=58), 17.1 yy (13-20) (SD=1.41)	ADHD, Conduct Disorder	MPH	NA	Cocaine dependence (n=29), Heroin dependence (n=23), Alcohol dependence (n=49), Cannabis Dependence (n=54), Other (n=7)	None	None	31% of patients have current ADHD diagnosis and 20% reported illicit drug diversion. One-third of entire patient population reported prior psychostimulant abuse; their initiation to alcohol or drug use generally occurred within a year of the ADHD diagnosis at almost 13 years of age. The 20% of patients diagnosed with ADHD also generally reported they initiated psychostimulant abuse after they had been diagnosed with ADHD.
Bruggisser et al. (26)	Retrospective case series (Switzerland)	N=14 (M=5; F=9), 31yy (17-51)	ADHD (n=4), Borderline Personality disorder (n=1)	MPH (only n=9/14 as prescribed)	MPH: n=9 oral, with doses ranging from 30 mg to 400 mg/day; n=1 nasal; and n=4 IV.	n=8 Polysubstance abuse (Alcohol, Benzodiazepines, Methadone, MPH, and Cocaine)	Symptoms and signs of sympathetic nervous stimulation (agitation, tachycardia, hypertension, anxiety, hallucination, headache, tremor and dizziness), epilepsy, and fever. Complications of parenteral injection of dissolved MPH tablets include local infection, cutaneous foreign body reactions, endocarditis of the tricuspid valve, pulmonary granulomatous disease and pulmonary hypertension	Sedative treatment, e.g., benzodiazepines; intravenous antibiotic treatment	Only 9 of the 14 MPH abusers (56%) had prescription for MPH. Two cases involved accidental intra-arterial injection and resulted in tissue necrosis leading to the amputation of a forearm and of fingertips, respectively. One patient presented to the emergency department because he needed a renewal of the prescription. One patient nasally administered 80 mg of MPH following intake of daily doses of 40–60 mg orally over three weeks while her prescribed dose was 20 mg/day. One patient took 270 mg of MPH orally with suicidal intentions. Two patients were monitored overnight in the intensive care unit due to agitation and epilepsy. Two patients were transferred to a psychiatric clinic following monitoring, including the suicidal patient.
Grau-López et al. (27)	Case Reports (Spain)	N=1, M	ADHD, Borderline Personality Disorder	MPH, Disulfiram (250 mg/day over 4 weeks)	MPH (2 weeks 36 mg/day up to 54 mg/day)	Alcohol dependence (60 to 100 g/day) and Cocaine dependence (2 g/week of cocaine)	Psychotic symptoms for 2 weeks (suspicion, self-reference, delusions of injury, auditory hallucinations, and delusional misconduct)	Olanzapine 10 mg/day	The patient was admitted to the hospital’s Detoxification Unit. At admission, patient drank 60 to 100g/day, consumed 2g/week of cocaine, and smoked 25 units/day. Toxicological history showed a cannabis dependence pattern for 5 years with sustained abstinence, alcohol dependence for 6 years, cocaine dependence for 10 years, and nicotine dependence for 16 years. Hypothetically, as MPH blocks the dopamine transporter and increases extracellular concentration of dopamine and disulfiram blocks the dopamine β-hydroxylase, increasing dopamine levels, the interaction between these 2 drugs can be related with the onset of psychotic symptoms.
Jaffe et al. (28)	Case Reports (USA)	N=1, M	ADHD, Dysthymic disorder	MPH	MPH Nasal (200 mg/day; tested positive at urine analysis)	Alcohol Use Disorder, CUD	Reduced attention span, low frustration tolerance, irritability, anxiety, talkativeness, sleep problems, feelings of loneliness, and suicidal thoughts	Individual and family therapy	Positive family history for drug addiction. Beside the MPH prescribed, an illicit use of large doses of prescribed MPH intranasally was recorded. He also began using LSD, which was not detected by urine testing and occasionally marijuana the day after his urine testing. The patient was treated in a dual diagnosis program, including 12-step program meetings as well as intensive individual and family therapy. He made good progress during a 32-day hospitalisation, and his depression resolved without the need for medication. One year follow-up revealed that he had abstained from drug use and had regularly attended Alcoholics/Narcotics Anonymous meetings
Lalanne et al. (29)	Case Report (France)	N=2, M 45 yy and 55 yy	1: Anxiety, Bipolar Disorder and Autism Spectrum Disorder 2: Asperger Syndrome, Anorexia, MDD and ADHD	1: Valproic acid to 2.5 g and lamotrigine to 400 mg (changed with 200 mg quetiapine per day) 2: Diazepam 20 mg per day and Acamprosate 900 mg per day (for alcohol dependence)	2: MPH 50 mg per day	1:Tobacco/Alcohol Dependence 2: Strong Black Tea Addiction (10 cups of tea every morning) and Alcohol Dependence	1: Early-morning waking, psychomotor retardation, weight loss, and excessive guilt. 2: A few hours after drinking this large quantity of tea, he felt anxious and complained of diarrhoea, tremors, and epigastric pain	1: Naltrexone for alcohol dependence (switched to baclofen, which was titrated over a 5- week period up to 170 mg per day) 2: sertraline (200 mg per day for 3 months), fluoxetine (40 mg per day for 6 months) and venlafaxine (250 mg per day for 1.5 months) for depressive episode and CBT	1: The patient has psychiatric and psychological outpatient appointments once and twice a week. Treatment for his mood disorder was 2 g valproic acid per day and 200 mg lamotrigine. To avoid delirium tremens, alcohol withdrawal was managed with 30 mg diazepam per day. He also received 900 mg acamprosate three times per day to prevent an alcohol relapse. He was regularly hospitalised for alcohol intoxication combined with behavioural disorders and depression. After a three-month stay in hospital, he returned home and continued his outpatient psychiatric and social care in our department, but he was regularly hospitalised for alcohol intoxication combined with behavioural disorders and depression. 2: The patient started CBT and remediation cognitive therapy and had a prescription for MPH. As the patient was afraid of becoming dependent on MPH treatment and highly anxious after taking it, he decided to take only 20 mg, and then decided to stop MPH. Both took psychostimulants to improve their neurocognitive abilities, and in both cases, their clinical picture was masked by their SUD, which resulted in a delayed diagnosis of their developmental illness

ADHD, Attention Deficit Hyperactivity Disorder; AMP, Amphetamine; AMPUD, Amphetamine Use Disorder; CBT, Cognitive Behavioural Therapy; CUD, Cannabis Use Disorders; MDD, Major Depression Disorder; DSM, Diagnostic and Statistical Manual of Mental Disorders; IR, immediate-release; IV, intravascular; MethAMP, Methamphetamine; MPH, Methylphenidate; MPHUD, Methylphenidate Use Disorder; NA, not available; OROS, Osmotic-release oral system; ROA, Route of Administration; SD, standard deviation; SUD, Substance Use Disorder.

Demographic findings showed a predominance of males (1,104/1,551). There were six studies reporting a mixed population (25, 26, 32, 33, 35, 36), and none in women alone. The age of the subjects ranged from 13 to 65 years. Apart for the diagnosis of ADHD, most important psychiatric diagnoses were mainly relating to conduct disorders (N=5) (31–34, 36), personality disorders (N=5) (25–27, 30, 35), major depression disorder (N=3) (29, 32, 34), anxiety disorders (N=3) (25, 34, 35), dysthymic disorder (N=1) (28), affective disorders (N=1) (35), autism spectrum disorder (N=1) (29), oppositional defiant disorder (N=1) (34), schizophrenic spectrum disorder (N=1) (35), anorexia nervosa (N=1) (29) and both psychotic and mood disorders (N=2) (25, 29).

The prevalence of psychiatric/neurological symptoms is reported in **Table 2**.

**Table 2:** Prevalence of the main psychiatric/neurological symptoms.

Psychiatric/neurological symptoms	Prevalence (N of patients where symptoms were reported/Total N of patients involved)	Reference
Craving	579/1551	Bron et al. (25); Konstenius et al. (30); Vogel et al. (35); Winhusen et al. (32)
Anxiety	297/1551	Bruggisser et al. (26); Jaffe et al. (28); Konstenius et al. (30); Lalanne et al. (29); Vogel et al. (35); Winhusen et al. (32)
Sympathetic nervous stimulation	89/1551	Bruggisser et al. (26); Jaffe et al. (28); Konstenius et al. (30); Vogel et al. (35)
Hallucinations	69/1551	Bruggisser et al. (26); Grau-López et al. (27); Konstenius et al. (30)
Alterations in the level of consciousness	69/1551	Bruggisser et al. (26); Grau-López et al. (27); Konstenius et al. (30)
Agitation/violent behaviour	68/1551	Bruggisser et al. (26); Konstenius et al. (30)
Suicide/suicide attempt	3/15	Bruggisser et al. (26); Jaffe et al. (28); Konstenius et al. (30)

A history of polysubstance use was reported in eight articles (N=8), including cannabis, cocaine, amphetamines, ketamine, heroin, nicotine (**Table 3**).

**Table 3:** Summary of results related to the main substance abused.

Substance abused	Prevalence (N of patients where the substance abuse was reported/Total N of patients involved)	Route of administration	Polysubstance abuse	References
Alcohol	475/1551	Oral	Benzodiazepines, Methadone, Cocaine, Cannabis, Heroin, Tobacco, Strong Black Tea Addiction, Nicotine	Bruggisser et al. (26); Gordon et al. (33); Grau-López et al. (27); Jaffe et al. (28); Lalanne et al. (29); Tamm et al., (31); Szobot et al. (34); Wilens et al. (36)
Cannabis	371/1551	Oral	Cocaine, Alcohol, Heroin, Nicotine	Gordon et al. (33); Jaffe et al. (28); Tamm et al., (31); Szobot et al. (34)
Nicotine	343/1551	Oral	Alcohol	Bron et al. (25); Lalanne et al. (29); Szobot et al. (34)
Cocaine	68/1551	Oral, Intravenous	Alcohol, Benzodiazepines, Methadone, Heroin, Cannabis, Nicotine	Bruggisser et al. (26); Gordon et al. (33); Grau-López et al. (27); Szobot et al. (34); Vogel et al. (35)
Amphetamine	54/1551	NA	None	Konstenius et al. (30)
Heroin	23/15	NA	Alcohol, Cannabis, Cocaine	Gordon et al. (33)

Eight of these articles were related to alcohol use disorder (N=8) (26–29, 31, 33, 34, 36). Five of these articles were related to Cocaine Use Disorder (N=5) (26, 27, 33–35). Four of these articles were related to Cannabis Use Disorder (N=4) (28, 31, 33, 34). Three articles were related to nicotine dependence (N=3) (25, 29, 34). Heroin dependence was reported in one article (N=1) (33) as well as Stimulant Use Disorder (N=1) (30). Among the most commonly reported psychiatric/neurological symptoms, it is noteworthy that the appearance of anxious symptoms was reported in six articles (26, 28–30, 32, 35). Signs and symptoms indicative of sympathetic nervous system stimulation were documented in four studies (26, 28, 30, 35). Craving was noted in four other studies (25, 30, 32, 35). Hallucinations were reported in three studies (26, 27, 30). Altered levels of consciousness were described in three studies (26, 27, 30), while agitation and violent behaviour were observed in two studies (26, 30).

Concerning organic symptoms (**Table 4**), the most prevalent were gastrointestinal symptoms such as loss of appetite, weight loss, diarrhoea, epigastric pain, abdominal discomfort, and dry mouth (N=4) (29, 30, 32, 35), followed by cardiovascular events, including palpitations, hypertension, chest pain, and dizziness (N=4) (26, 30, 32, 35). Musculoskeletal symptoms, such as fatigue, muscle cramps, and psychomotor retardation, were reported in three studies (N=3) (29, 30, 35), while respiratory symptoms, including tachypnea, pulmonary hypertension, and pulmonary granulomatosis, were reported in two studies (N=2) (26, 35).

**Table 4:** Summary of results related to organic symptoms.

Organic symptoms	Prevalence (N of patients where the organic symptoms was reported/Total N of patients involved)	References
Gastrointestinal symptoms: loss of appetite, weight loss, diarrhoea, epigastric pain, abdominal discomfort, dry mouth	201/1551	Konstenius et al. (30); Lalanne et al. (29); Vogel et al. (35); Winhusen et al. (32)
Cardiovascular symptoms: palpitations, high blood pressure, chest pain and dizziness	108/1551	Bruggisser et al. (26); Konstenius et al. (30); Vogel et al. (35); Winhusen et al. (32)
Musculoskeletal symptoms: fatigue, muscular cramps, psychomotor retardation	75/1551	Konstenius et al. (30); Lalanne et al. (29); Vogel et al. (35)
Respiratory symptoms: tachypnoea, pulmonary hypertension, pulmonary granulomatosis	34/1551	Bruggisser et al. (26); Vogel et al. (35)

No fatalities were reported; however, three hospitalizations due to suicidal intentions were documented (26, 28, 30). Of these, two were associated with oral abuse of methylphenidate (26, 30), and one was linked to intranasal abuse of methylphenidate (28). Regarding the abuse of methylphenidate, the most common dosages ranged from 10 to 72 mg, with several cases reporting significantly higher amounts ranging from 100 to 400 mg (N=75/1,551) (N=4) (26, 28, 30, 35), being normal dosages up to 60mg/day.

In terms of methylphenidate formulations, both ER (N=3) (32, 34, 36) and IR (N=1) (25) variants have been reported in cases of abuse. The most common was the ER formulation associated with “increased energy and feeling of satisfaction” (35). The primary routes of administration observed were oral ingestion (N=30), intravenous injection (N=11) and intranasal (N=9), with a notable preference for 60% of individuals opting for oral routes (26, 34, 35).

## 4 Discussion

According to the results of the study, the misuse of methylphenidate in this population often involves its non-medical use (25, 26, 28, 29, 35, 36), non-conventional routes of administration (26, 28, 35) e.g., intranasal or intravenous, and polysubstance abuse to potentiate or modulate the effects of methylphenidate. Eight of the twelve articles reported cases of polysubstance use (25, 26 ,28, 29, 34–36), with methylphenidate often co-abused with alcohol, cocaine, cannabis, and nicotine. The most important SUD associated with misuse of methylphenidate is alcohol use disorder, reported in eight of the twelve studies (26–29, 31, 33, 34, 36), followed by cocaine use disorder, reported in five studies (26, 27, 33–35), and cannabis use disorder, found in four studies (28, 31, 33, 34). These findings suggest that methylphenidate misuse, particularly among individuals with dual diagnosis, is often associated with other substance dependencies. Moreover, recent research highlights an exponential increase in the prevalence of ADHD diagnoses, which in turn suggests that the number of individuals treated with methylphenidate will rise accordingly. The general public has become more aware of ADHD leading people to bring up their concerns to a physician, which in turn might prompt more numbers of people to be diagnosed and to prescribe the treatment (37). Additionally, Google Trends data indicate a marked increase in ADHD diagnoses since 2004, reflecting a growing recognition and awareness of the disorder (38). As a result, the potential for misuse of methylphenidate is expected to grow, posing a heightened risk within vulnerable populations such as those with dual diagnosis. This further emphasises the need for more robust prevention strategies, including better monitoring of prescriptions and improved patient education to mitigate these risks.

The misuse of methylphenidate among individuals with dual diagnosis appeared associated with several adverse clinical outcomes. Sympathetic nervous system stimulation, reported in four of the reviewed studies (26, 28, 30, 35), was frequently associated with cardiovascular complications, including hypertension, tachycardia, and cognitive impairment. Methylphenidate misuse has also been linked to the exacerbation of psychiatric symptoms such as anxiety, depression, and mood disorders, as well as an increased risk of addiction due to its reinforcing effects. Consistently, findings from Shellenberg et al. (2020) (39) indicate that methylphenidate misuse is associated with worsening anxiety and depressive symptoms in patients with co-occurring psychiatric disorders, thereby amplifying mood instability. Additionally, the reinforcing effects of methylphenidate linked to an increase in dopamine release, particularly in individuals with ADHD (40), elevates its addictive potential, especially in individuals predisposed to SUD. Similarly, reinforcing effects have been associated to other medications, if administered at high dosage or unconventional routes, e.g. bupropion, a norepinephrine-dopamine reuptake inhibitor antidepressant, has also shown reinforcing properties due to its ability to increase dopamine levels, which can lead to misuse and dependence (41).

**Figure 2:**
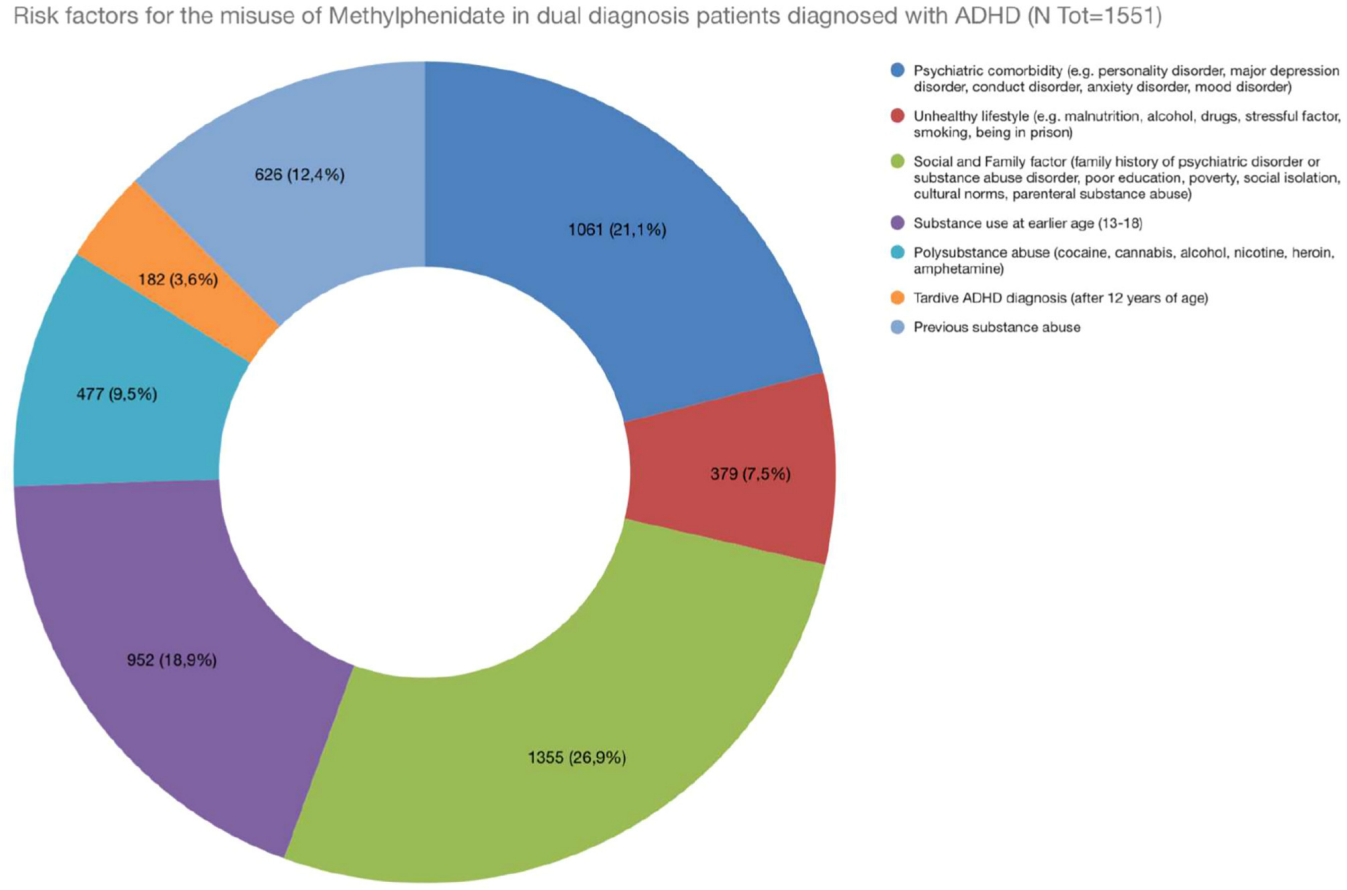
Risk factors for the misuse of Methylphenidate in dual diagnosis patients diagnosed with ADHD.

Several factors contributing to the increased risk of methylphenidate misuse among individuals with dual diagnoses have been identified (**Figure 2**). These include the severity of ADHD and psychiatric symptoms, a history of substance abuse, and various social and environmental influences such as peer pressure, easy access to medications, and stressful life events. Additional risk factors include early-onset substance abuse (ages 13-18), polysubstance abuse, late ADHD diagnosis, and sometimes preexisting mental health conditions and psychiatric comorbidities, particularly personality traits characterised by impulsivity and sensation-seeking, which are common in both ADHD and SUD, which may further increase the risk of future misuse. The analysis of risk factors associated with methylphenidate misuse revealed several key contributors. Indeed, social and family factors, such as a family history of psychiatric disorders, emerged as the most prevalent risk factor, with 1,355 patients in a population sample of 1,551 patients (25, 27–32, 34, 36). This was followed by psychiatric comorbidity, recorded in 1,061 patients, which includes diagnoses such as personality disorders, anxiety, and major depression, often exacerbating the likelihood of misuse. Interestingly, in the adolescent sample of patients, the most recorded diagnosis was a conduct disorder (31–34, 36), while among adults most common diagnoses were anxiety/mood disorders (25, 29), personality disorders (25–27, 30, 35) and schizophrenic spectrum disorder (35). Moreover, early substance use (13-18 years) was reported in 952/1,551 patients, highlighting how exposure to substances at a young age can significantly influence later patterns of substance abuse (25, 26, 30, 31, 33 ,36). Consistently, adolescent misuse of prescription stimulants is indeed associated with increased risk of later prescription drug (e.g. opioids and sedatives) misuse, This link is influenced by several factors, including the adolescent’s social environment, psychological stressors, and even genetic predispositions (42). Polysubstance abuse and unhealthy lifestyle choices (e.g., malnutrition and alcohol use) were still notable, affecting 477 and 379 patients, respectively. The unique environment of prisons, characterised by stress, limited access to healthcare, and a higher prevalence of substance use disorders, can exacerbate the potential for misuse. These patients may have a history of substance abuse and impulsive behaviour, increasing their vulnerability to misusing prescribed medications (30). This underscores the importance of monitoring and support for individuals with dual diagnosis in these settings to mitigate the risks associated with methylphenidate misuse.

Non-conventional routes of administration, e.g. intranasal, or intravenous, were recorded; these significantly influences the onset, intensity, and duration of effects, and overall the misuse pattern of methylphenidate. Indeed, if stimulants like methylphenidate are snorted or injected, patients may experience a rapid onset of euphoria, which can enhance their potential for misuse (43). According to the findings of the systematic review, both ER and IR formulations of methylphenidate were reported in cases of misuse; however it appears that ER formulations may be less prone to abuse due to their slower onset and longer duration of action. ER formulations were here associated with more controlled experiences of “increased energy” or “feelings of satisfaction,” whereas IR formulations were more often linked to rapid onset and intense effects, potentially increasing their misuse potential. IR formulations were often crushed and inhaled or injected for non-medical use, leading to a quicker and more potent euphoric effect. The slower onset and prolonged effects of the ER formulations made them less desirable for abuse (12, 44). Similar to the use of IR methylphenidate, IR quetiapine is associated with a higher risk of misuse due to the quick onset of effects that can lead to feelings of sedation and euphoria, making it more susceptible to abuse compared to its ER formulation (45). Both medications exhibit increased misuse potential due to their rapid effects, while their ER counterparts provide more stable and controlled therapeutic outcomes, reducing the likelihood of misuse.

### 4.1 Harm Reduction Strategies and Prevention of abuse/misuse of methylphenidate

Regulatory strategies play a critical role in preventing the misuse of medications. Integrating behavioural therapies alongside pharmacological treatment can enhance the effectiveness of prevention strategies. Cognitive-behavioural therapy (CBT) has been shown to reduce substance use providing patients with tools to resist the urge to misuse their medications. Open communication regarding potential side effects and misuse can empower patients to take an active role in their treatment reducing the likelihood of substance abuse. Multifaceted approach that includes education, screening, behavioural therapy, and collaborative care can significantly mitigate the risk of stimulant misuse and improve overall patient outcomes (46).

As highlighted by the Italian Medicines Agency (47), a global regulatory approach is essential to mitigate the phenomenon of drug abuse, particularly through coordinated efforts across different healthcare sectors. One of the key strategies reported was the implementation of Prescription Monitoring Programs (PMPs), which enable regulators and healthcare providers to monitor the prescribing of controlled substances, including stimulants, and assist in identifying patterns of overprescription. Such monitoring can prevent patients from obtaining multiple prescriptions, a known risk factor for abuse. Additionally, the implementation of Risk Evaluation and Mitigation Strategies (REMS) was reported, which are designed to ensure that the benefits of specific medications outweigh their associated risks. Education and awareness campaigns were also highlighted, aimed at increasing awareness of the risks related to the misuse of stimulant medications (48). The FDA recommends that healthcare providers counsel patients against sharing medications and monitor for signs of diversion. They have mandated updates to warning labels across stimulant medications to enhance awareness of misuse risks and to promote safe storage and disposal practices (49). The EUDA (European Union Drugs Agency) provides various guidelines and strategies aimed at harm reduction and prevention of drug misuse, particularly focusing on the diverse interventions necessary to tackle drug-related issues across Europe. One of the key frameworks established by the EUDA is the classification of prevention interventions into three main categories: universal, selective, and indicated. Universal prevention targets the entire population to deter or delay substance use onset, while selective prevention focuses on vulnerable groups at higher risk. Indicated prevention aims at individuals to prevent the development of substance dependence and reduce harmful use (50). Implementing these strategies can help mitigate the risks associated with stimulant medications, including methylphenidate.

## 5 Strengths and limitations of the study

This systematic review is pioneering in its investigation of the abuse and misuse of methylphenidate specifically within the context of dual diagnosis patients. By synthesising data from twelve original studies, this review highlights the significance of understanding the prevalence and characteristics of methylphenidate abuse, contributing valuable insights that are crucial for the prevention of SUD in this vulnerable population. The novelty of the findings underscores the urgent need for healthcare providers to recognize the potential risks associated with methylphenidate, particularly its misuse at high doses or through non-oral routes. Given the substantial health risks associated with methylphenidate abuse, including addiction and adverse health outcomes, the data presented in this study provide an essential foundation for developing strategies aimed at improving patient monitoring, regulatory measures, prevention and psychoeducation. However, certain limitations must be acknowledged. There is a potential for publication bias, as the review exclusively included studies published in English, which may have resulted in the exclusion of relevant research available in other languages. Moreover, the phenomenon of methylphenidate abuse may be underestimated, particularly in cases with unclear or incomplete medical histories, where detection challenges could lead to under-recognition of the issue. These factors may limit the generalizability of the findings. Nonetheless, the insights derived from this review pave the way for future research and clinical practices, highlighting the need for careful monitoring and regulation of methylphenidate prescriptions in dual diagnosis patients to mitigate risks effectively.

## 6 Conclusion

While it is an effective treatment for ADHD and other conditions, methylphenidate potential for abuse, particularly at high doses or via non-oral routes, in dual diagnosis patients underscores the importance of careful monitoring and regulation. The misuse of methylphenidate is associated with substantial risks, including addiction, adverse health outcomes, and broader public health concerns. While it is an effective treatment for ADHD, methylphenidate’s potential for abuse, particularly at high doses or via non-oral routes, in dual diagnosis patients underscores the importance of careful monitoring and regulation. The misuse of methylphenidate is associated with substantial risks, including addiction, adverse health outcomes, and broader public health concerns. To mitigate these risks, it is crucial to prioritise the use of extended-release (ER) formulations in dual diagnosis patients, as they offer a lower potential for abuse. Additionally, increasing the frequency of patient monitoring visits can help identify and address early signs of misuse. Effective psychoeducation at the beginning of treatment is also essential to ensure patients are well-informed about the risks and safe use of the medication. This should also include clear communication regarding the importance of adhering to prescribed doses increasing the awareness about the potential dangers of misuse. Addressing these risks requires a comprehensive approach, including stricter prescription monitoring, patient education about the risks of misuse, and more effective regulatory controls to prevent diversion in this vulnerable population.

## Data Availability

The original contributions presented in the study are included in the article/**Supplementary Material**. Further inquiries can be directed to the corresponding author.
