# Supplementary material for: Methylphenidate abuse and misuse in patients affected with a psychiatric disorder and a substance use disorder: a systematic review
Source: Front Psychiatry. 2024 Nov 18;15:1508732. doi: 10.3389/fpsyt.2024.1508732 (PMC11609911; doi:10.3389/fpsyt.2024.1508732)
Supplement: Supplementary file 2 [file DataSheet2.pdf]

AMSTAR 2: a critical appraisal tool for systematic reviews that include randomised or non-randomised studies of healthcare interventions, or both

Risk of Bias Assessment Table

Bias Type	Leanne Tamm_2013	Maija Konstenius_2014	Theresa M Winhusen_2011	Claudia M Szobot_2008	Tannetje I Bron_2013	Marc Vogel_2016	Wilens, Timothy E._2006	Gordon, Susan Merle_2004	Marcel Bruggisser_2011	Lara Grau-López_2012 (ROBINS-I)	S L Jaffe_1991	Lalanne, Laurence_2015
Randomization Bias (Selection Bias)	Low risk of bias	Low risk of bias - central randomization with block randomization, independent handling by pharmacy	Low risk of bias - Participants were randomized to OROS-MPH or matching placebo in a 1:1 ratio, stratified by site, and completed by computer at a centralized location. This method of randomization is typically robust, minimizing selection bias.	Moderate risk of bias -	Low risk of bias	Moderate risk of bias	Moderate risk of bias	Moderate risk of bias	Moderate risk of bias	Unclear risk of bias	Unclear risk of bias	Unclear risk of bias
Blinding of Participants and Personnel (Performance Bias)<br><br>Selection of Partecipants	Low risk of bias	Low risk of bias - double-blinding maintained throughout trial	Low risk of bias - The study employed a matching placebo, indicating that both participa	High risk of bias - absence of blinding	Moderate risk of bias	Moderate risk of bias	Moderate risk of bias	Low risk of bias	Low risk of bias	Moderate risk of bias	High risk of bias	Moderate risk of bias

			nts and study staff were likely blinded to group assignments. This helps mitigate performance bias.									
Incomplete Outcome Data (Attrition Bias)	Low to moderate risk of bias	Moderate risk of bias - high attrition rate; attempts to account for dropout bias by imputing missing data	Moderate risk of bias - pending more information on attrition rates and handling of dropouts.	Unclear risk of bias	Moderate risk of bias	Low risk of bias	Low risk of bias	Low risk of bias	Moderate risk of bias	Low risk of bias	Moderate risk of bias	Unclear risk of bias
Selective Reporting (Reporting Bias)	Low risk of bias	Low risk of bias - all pre-specified outcomes appear to be reported	Moderate risk of bias - due to potential selective reporting and limitations in the measures used.	Moderate risk of bias	Moderate risk of bias	Moderate risk of bias	Moderate risk of bias	Moderate risk of bias	Moderate risk of bias	Moderate risk of bias	Moderate risk of bias	Moderate risk of bias
Bias Due to Reported Limitations	Moderate risk of bias	Moderate risk of bias - small sample size and unmeasured dropout behaviors may affect reliability	Moderate risk of bias - limitations may influence the interpretation and generalizability of the results.	Moderate risk of bias	Low risk of bias	Moderate risk of bias	Moderate risk of bias	Moderate risk of bias	Moderate risk of bias	Moderate risk of bias	Moderate risk of bias	Moderate risk of bias

AMSTAR 2: a critical appraisal tool for systematic reviews that include randomised or non-randomised studies of healthcare interventions, or both

**1. Did the research questions and inclusion criteria for the review include the components of PICO?**	Yes<br>Yes<br>Yes<br>Yes<br>Yes
**2. Did the report of the review contain an explicit statement that the review methods were established prior to the conduct of the review and did the report justify any significant deviations from the protocol?**	YesYesYesYesYes
**3. Did the review authors explain their selection of the study designs for inclusion in the review?**	Yes<br>Yes
**4. Did the review authors use a comprehensive literature search strategy?**	Yes<br>Yes<br>Yes<br>Yes<br>Yes<br>Yes<br>Yes<br>Yes<br>Yes

AMSTAR 2: a critical appraisal tool for systematic reviews that include randomised or non-randomised studies of healthcare interventions, or both

**5. Did the review authors perform study selection in duplicate?**	Yes<br>Yes
**6. Did the review authors perform data extraction in duplicate?**	Yes<br>Yes
**7. Did the review authors provide a list of excluded studies and justify the exclusions?**	Yes<br>Yes<br>Yes
**8. Did the review authors describe the included studies in adequate detail?**	Yes<br>Yes<br>Yes<br>Yes<br>Yes<br>Yes<br>Yes<br>Yes<br>Yes<br>Yes<br>Yes
**9. Did the review authors use a satisfactory technique for assessing the risk of bias (RoB) in individual studies that were included in the review?**<br>**RCT**	Yes
**NRSI**	Yes<br>Yes<br>Yes
**10. Did the review authors report on the sources of funding for the studies included in the review?**	Yes<br>Yes
**11. If meta-analysis was performed did the review authors use appropriate methods for statistical combination of results?**<br>**RCT**	0
**NRSI**	0
**12. If meta-analysis was performed, did the review authors assess the potential impact of RoB in individual studies on the results of the meta-analysis or other evidence synthesis?**	0

AMSTAR 2: a critical appraisal tool for systematic reviews that include randomised or non-randomised studies of healthcare interventions, or both

**13. Did the review authors account for RoB in individual studies when interpreting/ discussing the results of the review?**	Yes<br>Yes
**14. Did the review authors provide a satisfactory explanation for, and discussion of, any heterogeneity observed in the results of the review?**	Yes<br>Yes
**15. If they performed quantitative synthesis did the review authors carry out an adequate investigation of publication bias (small study bias) and discuss its likely impact on the results of the review?**	Yes<br>Yes
**16. Did the review authors report any potential sources of conflict of interest, including any funding they received for conducting the review?**	Yes<br>Yes
