# Supplementary material for: Methylphenidate abuse and misuse in patients affected with a psychiatric disorder and a substance use disorder: a systematic review
Source: Front Psychiatry. 2024 Nov 18;15:1508732. doi: 10.3389/fpsyt.2024.1508732 (PMC11609911; doi:10.3389/fpsyt.2024.1508732)
Supplement: Supplementary file 3 [file Table1.docx]

**Table 1. Supplementary materials: PICO Table**

|  | P  (Patient or Population or Problem) | I  (Intervention, prognostic factor, exposure) | C  (Comparison | O  (Outcome) | Research Question |
| --- | --- | --- | --- | --- | --- |
| Intention | Patients with a dual diagnosis (co-occurring psychiatric disorder and substance use disorder) misusing and abusing of methylphenidate | Rate of methylphenidate abuse/misuse in a specific population of patients | General population | Understanding the prevalence and pattern of abuse/misuse of methylphenidate among patients with dual diagnosis (concurrent psychiatric disorder and substance use disorder), and what are the associated clinical outcomes and risk factors | What are prevalence and patterns of methylphenidate abuse or misuse among patients with dual diagnosis, and what are the associated risk factors and clinical outcomes? |
